# The *labial* gene is required to terminate proliferation of identified neuroblasts in postembryonic development of the *Drosophila* brain

**DOI:** 10.1242/bio.020230

**Published:** 2016-07-13

**Authors:** Philipp A. Kuert, Bruno C. Bello, Heinrich Reichert

There were two errors published in *Biol. Open* **1**, 1006-1015.

1. Nomenclature: The lineages as previously published in Pereanu and Hartenstein (2006) should have been TRdl instead of TRld, and TRvl instead of TRlv. This affects all instances in the text as well as Figs 2; 3A-D; 4A,B,E,F; 6C,K; 7A,B,E,F and 8, and Fig. S1.

2. The late embryonic stage mentioned on pages 1012, 1014, and in Fig. 8 and Fig. S2 should have been stage 16 and not stage 17.

These errors do not affect the conclusions of the paper.

The authors apologise to the readers for any confusion that these errors might have caused.

